# Therapists’ Views of Mechanisms of Change in Psychotherapy: A Mixed-Method Approach

**DOI:** 10.3389/fpsyg.2022.565800

**Published:** 2022-04-14

**Authors:** Dana Tzur Bitan, Shani Shalev, Shiran Abayed

**Affiliations:** ^1^Department of Behavioral Sciences, Ariel University, Ariel, Israel; ^2^Shalvata Mental Health Center, Hod Hasharon, Sackler School of Medicine, Tel Aviv University, Tel Aviv, Israel

**Keywords:** psychotherapy, process research, process expectations, therapists, change mechanisms

## Abstract

The question of what works in psychotherapy has been a subject of debate in the recent years, occupying both clinicians and researchers. In this study, we aimed to assess the current perspectives held by clinicians regarding the processes which produce changes in psychotherapy, as well as the predictors of specific views. Licensed therapists (*n* = 107), consisting mainly of psychodynamically and integratively oriented psychologists, were asked to write in their own words what they think works in psychotherapy. Thematic analysis was employed to assess the main mechanisms of change as perceived by the therapists. Differences in the prevalence of specific themes were assessed using Friedman and Wilcoxon signed-rank tests. Univariate logistic regressions were employed to assess the factors that predict the probability of reporting a specific mechanism of change. The results indicated that the therapeutic bond was the most highly reported mechanism of change, followed by theory-driven mechanisms of change, therapist characteristics, therapist professionalism, and client motivation. Male therapists were more likely to indicate the professionalism as a mechanism of change compared to female therapists. Higher education was associated with lower reports of therapists’ characteristics as the mechanisms of change. These results suggest that therapists acknowledge the importance of the working alliance, and that the perception of the mechanism of change is associated with various factors which comprise therapist orientation. Limitations and directions for future research are discussed.

## Introduction

Many studies have assessed the effect of patients’ expectations on the therapeutic process and outcome. These studies suggest that patients’ expectations are an integral component in the course and outcome of psychotherapy, which estimated to account for 15% of the variance in the outcome (Norcross and Lambert, 2011). Furthermore, the studies indicate that congruence between patients’ expectations and actual treatment is associated with important clinical factors, such as early engagement in therapy (Elkin et al., 1999), the development of the working alliance (Iacoviello et al., 2007), and outcome (Swift and Callahan, 2009). This congruence is likely to be associated with both patients’ and therapists’ expectations of the therapeutic process. Nonetheless, while patients’ expectations have been extensively studied, less is known about therapists’ expectations, and specifically, what they expect will transpire in a meaningful therapeutic process.

The exploration of therapists’ expectations regarding the processes of change may enable a clear identification of potential gaps between patients’ and their therapists’ expectations and can set the stage for subsequent studies that assess the effect of such gaps on therapy outcome. Nonetheless, therapists’ attitudes and perceptions have been relatively under-studied in the recent years. The studies investigating therapists’ views and perceptions have typically focused on the therapists’ attitudes toward specific interventions or approaches, such as the use of homework in cognitive-behavioral therapy (CBT) (Kazantzis et al., 2005), the utility of self-disclosure (Carew, 2009), or the implementation of empirically based treatments (Gaudiano et al., 2011). A very few studies have addressed therapists’ process perceptions and their effect on therapeutic process and outcome. In a systematic literature review, Lingiardi et al. (2018) reported on three early studies that assessed therapists’ attitudes and value systems and suggested that these attitudes had an effect on the outcomes of long−term psychoanalysis (Lafferty et al., 1989; Berghout and Zevalkink, 2011) and on treatment continuation (Frank et al., 1987). Although they concluded that these attitudes had a weak effect on outcome, they also pointed to the lack of updated studies in this area of research. Thus, it seems that the issue of therapists’ attitudes and expectations is in need of additional research that takes recent developments in the field of psychotherapy research into account. These developments mostly include recent findings of the effects of moderators and mediators on therapy outcomes, as well as recent developments in the study of mechanisms of change (Falkenström et al., 2017).

The study of mechanisms of change in psychotherapy has produced a large body of knowledge and also several potential mediators, which may play a crucial role in causing a therapeutic change. Examples of such mechanisms include the working alliance (Flückiger et al., 2018), transference interpretations (Høglend et al., 2008), the achievement of insight (Kallestad et al., 2010), and so on. In parallel with these developments, there has been an ongoing debate regarding the question of whether common factors – vs. more specific, ingredient-oriented factors – are the main mechanisms of change (Wampold and Imel, 2015). Thus, a question that arises is whether this debate is reflected in therapists’ views of the mechanisms of change, and whether different therapists have different views on the main therapeutic process that should transpire to produce therapeutic change. In a recent study conducted by Tzur Bitan and Abayed (2020), therapists, patients, and lay individuals with no prior experience in psychotherapy reported their process expectations using a designated measure aimed to measure process expectations. They found that therapists rated emotional processing and patient–therapist relationships as higher in importance than did patients and lay individuals, but patients and lay individuals rated cognitive and emotional reconstruction higher than did therapists. Nonetheless, as the study of therapists’ process expectations is relatively limited, there is a need to further assess therapists’ process expectations using both nomothetic and idiographic approaches.

In this study, we explored therapists’ views and perceptions regarding the mechanisms of change, while employing a mixed-model approach, which comprises both qualitative and quantitative analyses. Therapists consisting mainly of psychodynamically and integratively oriented psychologists were requested to freely write what they think works in psychotherapy. A thematic analysis was utilized to obtain the main themes which reflect the expressed mechanisms of change suggested by the therapists. These themes were then combined into general process factors and assessed for prevalence, rankings, and associated demographic and clinical factors.

## Materials and Methods

### Participants

Inclusion criteria for the therapist sample were as follows: (a) a professional license to practice psychotherapy and (b) the ability to respond to online assessments *via* a computerized platform. The participants were 107 licensed therapists. Descriptive information regarding the therapist sample is presented in Table 1. The mean age of the therapists was 36.44 (SD = 6.40) and it ranged from 26 to 61. The majority were women (72%) and Israeli-born (86.9%). Most comprised psychologists (95.3%), with 53.3% of them being interns and 46.7% being senior psychologists. It should be noted that in Israel, interns can only begin their internships after being licensed by the Ministry of Health as certified psychologists. A license to practice psychology is granted after 2 years of theoretical and clinical supervised work, which is then followed by 4 additional years of internship. After these 4 years, psychologists are licensed as the senior psychologists by the Ministry of Health. About 31.8% of the sample reported their main place of work to be a general hospital or private clinic (24.3%). Among them, the majority identified as integrative in their psychotherapy orientation (56.1%), followed by psychodynamic (39.2%). Only 4.7% of the therapist sample identified themselves as cognitive-behavioral oriented.

**TABLE 1:** Characteristics of the study sample (*n* = 107).

Characteristics	Psychotherapists (*n* = 107)
Age (Mean, SD)	36.44 (6.40)
**Sex**	
Male	30(28%)
Female	77(72%)
**Country**	
Israel	93(86.9%)
Other	14(13.1%)
Education (Mean, SD)	18.2 (2.121)
**Training stage**	
Internship	57(53.3%)
Senior psychologist	50(46.7%)
**Type of training**	
Psychologist	102(95.3%)
Psychiatrist	0
Social worker	5(4.7%)
**Main work**	
Private clinic	26(24.3%)
Psychiatric hospital	12(12.1%)
General hospital	34(31.8%)
Outpatient clinic	13(12.1%)
Other	21(19.6%)
**Specialty**	
Rehabilitative psychologist	43(40.2%)
Clinical psychologist	38(35.5%)
Health psychologist	15(14%)
Educational psychologist	3(2.8%)
Developmental psychologist	3(2.8%)
**Psychotherapist orientation**	
Psychodynamic	42(39.2%)
Cognitive-behavioral	5(4.7%)
Integrative	60(56.1%)
Perceived knowledge of what works (Mean, SD)	6.88 (1.40)

### Measures

#### Open-Ended Question Regarding Active Processes of Psychotherapy

To facilitate an open response regarding the perceived mechanism of change, participants were requested to write freely about their views of the active processes that they perceived to produce a meaningful change in psychotherapy. The following question was presented to therapists to facilitate a free response: “In this part, we would like to ask you to write in your own words: What do you think should happen in psychotherapy in order to achieve a meaningful psychological change? Please try to elaborate as much as possible.” Therapists were also asked to what extent they were knowledgeable about the mechanisms of change in psychotherapy, that is, to what extent they know what works in psychotherapy. Responses were rated on a Likert scale ranging from 1 (*not at all*) to 7 (*very much*).

### Procedure

A qualitative assessment of therapists’ process expectations was conducted as a part of a larger study that assesses differences between therapists, lay individuals, and individuals previously engaging in psychotherapy, in their views of what works in psychotherapy (Tzur Bitan and Abayed, 2020). The study was approved by the institutional review board of the university conducting the study. Therapists were recruited *via* professional listservs and volunteer sampling conducted in clinics and public hospitals in a snowball manner, which might have led the sample to consist primarily of professionals from the discipline of psychology. Individuals who were agreeing to participate signed informed consent. Upon agreeing to participate, therapists were asked to answer the open-ended question regarding the perceived mechanism of change, as well as to provide demographic details and information about their clinical work and training. Blank lines were provided for the typing of written answers. The survey was sent using a link of the Qualtrics Research Suite Platform, and therapists freely provided their answers for the specified questions.

### Statistical Analyses

The Braun and Clarke (2006) approach to thematic analysis was employed to assess the main mechanisms of change provided by the therapists’ responses. The Braun and Clarke (2006) approach includes a six-phase guide to data coding into themes through iterative cycles of reading, coding, defining, and summarizing. The themes were coded by two independent undergraduate students, two graduate students, and by a PhD-level scholar. The excerpts were initially reviewed by two members of the research team. A list of themes was then formulated by each member separately. These lists were then unified, and common themes that were observed by both members were retained and redefined following discussions with the research group members. Inter-rater reliability was assessed by a qualitative comparison of the general themes produced by the two independent coders, made by a third researcher. To assess differences in the prevalence of specific themes, a Friedman chi-square test was employed, followed by a Wilcoxon signed-rank test to assess the origins of differences. Finally, a set of univariate logistic regressions was employed to assess the probability of reporting a specific theme as a function of demographic and clinical variables. Odds ratios (ORs) were performed with a 95% confidence interval, and *p*-values were corrected for multiple comparisons using the Bonferroni correction. Statistical analyses were performed using the Statistical Package for the Social Sciences (SPSS) for windows v.24 (IBM Corp., Armonk, NY).

### Researcher Positionality

The study was initiated by the first author (DTB) and conducted by a graduate student (SA) as a secondary analysis of a doctoral dissertation submitted to the Department of Behavioral Science at the university with which the authors are affiliated. The incentive to perform the study was the need to map potential ingredients of therapeutic change, as viewed not only by the empirical literature, but also from the perspective of practicing therapists. To make sure authors were not biased toward specific mechanisms of change, the raters (SS) performing the initial independent screen of excerpts were undergraduate students with no prior clinical training (which might have biased them toward a specific theme). After formulating a unified list of themes, these themes were reviewed and redefined by the two graduate students and finalized by the first author. Researchers’ background included one practicing therapist specializing in crisis-focused psychotherapy and primarily psychodynamically oriented; one postgraduate student practicing therapy as a part of training provided by an integratively oriented (combining both cognitive-behavioral and psychodynamic approach) clinical staff; and one postgraduate student who was during her undergraduate studies when conducting in the study. Our hypotheses, which were driven by the prior studies conducted by this research groups, were that therapists will point to the patient–therapist relationship as an essential ingredient needed to achieve a meaningful psychological change.

## Results

Overall, the therapists reported 370 different responses related to mechanisms expected to facilitate change following psychotherapy (*M* = 3.44, SD = 1.54). A list of all of the themes expressed by the therapists as the potential mechanisms of change is reported in Supplementary Appendix A. Overall, the responses were divided into 53 different themes. Table 2 presents the 10 most cited themes, as well as frequencies and examples of each theme.

**TABLE 2:** List of the 10 *most expressed* themes by therapists as the mechanisms of change in psychotherapy, number of responses, and percentages of the overall number of responses.

Theme	n	%	Examples
Therapeutic bond	82	22.16%	“To evoke change there should be a strong therapeutic bond between the therapist and the patient”; “The main work is conducted within the therapeutic bond between the therapist and the patient. The ability to talk about the bond, evolve, and go through struggles within it”
Trust	32	8.65%	“Sense of security and trust in the relations”; “Trusting relations that enable the expression of complex contents in the room and the processing of these contents”
Empathy	21	5.68%	“Therapist should be empathic to the difficulties arising in the therapeutic process”; “An empathic bond should be formed to allow for expression of a broad range of emotions”
Introspection	18	4.86%	“When the person gets to know the inner processes occurring within him/her and how these processes affect and shape his/her self-image, his/her thinking processes, his/her relations”; “The introspection and expansion of awareness of feelings and emotions”
Secure environment	17	4.59%	“An open space for the patient to feel secure, hugged, and accepted”; “The patient should have a secure, non-judgmental, and accepting space, which enables him/her to bring him/herself and introspect”
Motivation	15	4.05%	“Motivation and willingness of the patient to meet him/herself in complex, dark, and unfamiliar places”; “Being ready and motivated for a change”
Insight	12	3.24%	“Insight toward factors which facilitate pain and create a sense of being stuck”; “Having insights about the coping strategies, self-perception, and relations of the patient”
Theoretical knowledge	9	2.43%	“The treatment should be adapted to the patient in terms of the theoretical and practical knowledge of the therapist and the interventions used”; “Theoretical knowledge about diagnoses, patient personality, treatment approaches”
Transference interpretations	9	2.43%	“Appropriate management of the counter-transference”; “Change in the transference and counter-transference and internalization of the therapist”; “Being attentive to the transferential relations, to the enactment”
Defining goals	8	2.16%	“Defining the therapy as set by the goals defined by the patient and the therapist collaboratively”; “The treatment should have clear goals, which will be openly defined and discussed by both the patient and the therapist, and the treatment will be aimed toward achieving this goal”

*Percentages were calculated from the overall number of excerpts (n = 364). Only the primary themes were listed in Table 2; the full list can be obtained from the first author.*

Themes were then clustered into factors by their content commonalities, with themes expressing common thematic mechanisms unified into one cluster. This process resulted in a total of eight general factors. The first factor was labeled “Therapeutic bond” and included themes such as openness, trust, open communication, having a secure environment, and devotion. The second factor was labeled “Therapist characteristics” and included themes such as empathy, curiousness, flexibility, patience, humbleness, and passion. The third factor was labeled “Professionalism” and included elements such as theoretical knowledge, professionalism, setting, defining goals, and knowing treatment approaches. The fourth factor was labeled “Theory-driven mechanisms” and included elements such as object relations, transference interpretations, defense mechanisms, validation, and containment. The fifth factor was labeled “Client motivation” and included elements such as willingness to change, motivation, and readiness for change. The sixth factor was labeled “Client characteristics” and included themes such as flexibility, beliefs and expectations, patience/sustainability, curiousness, mental resources, and matureness. The seventh factor was labeled “Cognitions” and included elements such as a change in thought, psychoeducation, and adaptive cognitions. The eighth factor was labeled “Unable/general factors” and included general elements such as personality change, understanding the client, integrability, or unable to answer. Table 3 presents the eight factors, which summarize the themes expressed by the therapists, as well as percentages of responses from the total number of therapists (how many therapists provided a specific response) and from the total number of responses (how many responses expressed a specific theme from the overall sum of responses). It should be noted that when several responses were related to one factor, these responses were counted as one, which results in the total sum being lower than the overall number of responses. For example, if a therapist reported both openness and trust in his/her excerpts, these two themes were clustered together as a response to the “therapeutic bond” factor.

**TABLE 3:** Percentages of responses within therapists and within total number of responses, mean ranks, and significance of differences in rankings.

Factor number	n	% of the total therapists	% of the total responses	Mean rank	Significantly larger rank than factor
1 Therapeutic bond	95	88.78%	36.54%	7.78	2–8
2 Therapists’ characteristics	29	25.23%	11.15%	5.00	7–8
3 Professionalism	26	24.29%	10.00%	4.88	7–8
4 Theory-driven mechanism	55	51.40%	21.15%	6.10	3,5–8
5 Clients’ characteristics	14	13.08%	5.38%	4.37	
6 Clients’ motivation	26	24.29%	10.00%	4.88	7–8
7 Cognition	5	4.67%	1.92%	4.00	
8 Unable/General statements	10	9.30%	3.84%	3.95	

*Factors are numbered by their clustering; see Supplementary Appendix A for full list of factors and associated themes. A Bonferroni correction was set with a significance level of 0.0013.*

To assess the difference in the ordering of the responses, a Friedman chi-square test was utilized. The results indicated a statistically significant difference in the overall rankings of the eight factors [Friedman chi-square (Friedman’s Q) = 316.40, df = 8, *p* < 0.001]. A Wilcoxon signed-rank test was then performed to assess the origins of the differences, while applying a Bonferroni correction for 36 comparisons, which yields a *p*-value of 0.0013. The results indicated that the therapeutic bond was ordered the highest of all factors, followed by theory-driven mechanisms and therapists’ characteristics. Professionalism and client motivation then followed and were ranked the same, followed by the clients’ characteristics.

Univariate logistic regressions were employed to explore the association between various variables related to therapists’ characteristics and the expression of one of the eight content factors. Results are presented in Table 4.

**TABLE 4:** Association between the content factors of the mechanisms of change and therapists’ characteristics.

Factor	Gender^a^				Education				Experience^b^			
	OR	95% CI		p	OR	95% CI		P	OR	95% CI		p
1	2.00	0.58	6.87	0.27	0.87	0.68	1.11	0.27	0.59	0.17	1.99	0.39
2	1.31	0.49	3.50	0.58	**0.69**	**0.51**	**0.94**	**0.02**	0.50	0.20	1.21	0.12
3	**0.21**	**0.08**	**0.54**	**0.001**	1.12	0.9	1.37	0.25	1.18	0.49	2.88	0.70
4	1.08	0.46	2.51	0.85	1.15	0.94	1.41	0.15	1.64	0.76	3.54	0.20
5	0.66	0.20	2.16	0.49	1.22	0.97	1.53	0.08	1.16	0.37	3.57	0.79
6	0.66	0.25	1.70	0.39	0.91	0.72	1.15	0.45	0.97	0.40	2.35	0.97
7	3.83	0.46	31.69	0.21	0.83	0.58	1.20	0.33	1.75	0.28	10.95	0.54
8	1.58	0.17	14.82	0.68	1.08	0.74	1.57	0.66	1.15	0.31	4.25	0.82

*^a^Reference group is male. ^b^Reference group is intern.*

As can be seen in Table 4, experience (being either an intern or senior psychologist) did not predict any of the themes reported by the therapists. Gender was found to be significantly associated with Factor 3, which indicates that men were more likely to indicate professionalism as a mechanism of change. The years of education was associated with Factor 2, which indicates that higher education was associated with lower reports of therapists’ characteristics as the mechanisms of change. There was also a trend of association between education and Factor 5 (*p* = 0.08), which indicates that higher education was associated with higher reports of clients’ characteristics as the mechanisms of change.

## Discussion

In this study, we aimed to explore therapists’ spontaneous responses to the question of what works in psychotherapy. The analyses indicated that therapists have many varied ideas and expectations regarding the mechanisms producing therapeutic change. Nonetheless, the results indicated that most therapists (88.7%) prioritized elements of the therapeutic bond in their responses. The results further demonstrated that the second-ranked group of responses were those related to a specific theory, followed by the responses related to the therapists’ characteristics, professionalism, and clients’ motivation.

The therapeutic bond, a central component of the therapeutic alliance, is considered as one of the most prominent and reliable common factors in psychotherapy (Orlinsky et al., 2004; Muran and Barber, 2010; McAleavey and Castonguay, 2015). Although this finding is well-acknowledged in the psychotherapy research community, less is known about the knowledge of therapists practicing in the clinical field. There have been relatively few studies that examined therapists’ perceptions regarding the importance of the alliance, and most of them were either in the context of a different research question, or only quantitatively studied. Tzur Bitan and Abayed (2020) used a quantitative measure of process expectations to evaluate differences between therapists and patients. They reported that when asked to numerically rank (in descending order) the expected active processes of change, therapists ranked the establishment of positive patient–therapist relations as more important than did patients and lay individuals. These results were also confirmed in the quantitative and qualitative analyses of the therapists’ verbal responses, which demonstrates therapists’ view of the therapeutic process as “the ability to talk about the bond, evolve, and go through struggles within it.”

A total of two quantitative studies were previously performed to evaluate therapists’ perceptions toward research, which also reported on therapists’ views of the alliance. Pagoto et al. (2007) asked the members of professional listservs to nominate the top barriers and facilitators to use the evidence-based practice and found that clinicians felt that research might potentially degrade the more human aspects of therapy such as empathy, creativity, and the working alliance. Similarly, Gyani et al. (2015) interviewed 33 therapists in the United Kingdom regarding their attitudes toward research and found that all therapists apart from one stated that the therapeutic alliance was the main determinant of success for treatment. Using a more quantitative approach, Larsson et al. (2010) assessed the psychometric properties of a questionnaire developed to inquire about the perceptions of how psychotherapy ought to be pursued among Swedish therapists. They found that items related to common factors, which include the alliance, had the highest mean score among therapists, with no significant difference between CBT and psychodynamically oriented therapists. Thus, the results of our qualitative assessment support the idea that therapists view the alliance as an important ingredient of the therapeutic process.

The investigative analyses inquiring into factors that might predict the significance of one mechanism or the other indicated that male therapists viewed professionalism as a mechanism of change to a higher degree than did female therapists, and that therapists with more years of education were less likely to view therapists’ characteristics as a mechanism of change. The predictive effect of gender on attitudes toward psychotherapy has been previously reported by Larsson et al. (2010), who found that female therapists viewed common factors as more important than did male therapists. The results of the current analyses support the effect of gender on therapists’ perceptions, by suggesting that male therapists place more importance on more structured elements of psychotherapy, such as setting, theoretical knowledge, and familiarity with treatment approaches. The finding in which more educated therapists tended to give less weight to therapists’ characteristics as the mechanisms of change can be associated with the effects of experience and therapeutic training. It has been shown that therapists have attributional views regarding the role of the therapist vs. the client (Murdock et al., 2010), and that most therapists highlight patients’ recognition of competence at therapy termination (Norcross et al., 2017). Thus, it can be hypothesized that training and experience cause therapists to give less weight to their own interventions and place more importance on the clients’ actions. This hypothesis should be subjected to future research.

The findings of this study have several potential clinical and empirical implications. The studies indicate that value systems and attitudes of therapists can predict therapy outcomes (Berghout and Zevalkink, 2011; Lingiardi et al., 2018). Experimental studies also show that therapeutic processes benefit from interventions aimed at bridging gaps in expectations of the therapy process (Guajardo and Anderson, 2007). Thus, there is an ongoing need to continue to study the evolution of therapists’ perceptions regarding mechanisms of change as this area of research continues to develop. Therapists in this study reported a mean of 6.88 (in a scale of 1 to 7) when asked to what extent they know what works in psychotherapy. This finding is contrasted with current views in psychotherapy research, which generally call to further investigate the processes leading to therapeutic change (Zilcha-Mano and Ramseyer, 2020). Nonetheless, the fact that therapists indicated the therapeutic bond as the mechanism of change most frequently resonates well with current findings, which point to the working alliance as one of the strongest predictors of therapeutic success (Martin et al., 2000), and indicated that therapists are updated with current empirical views.

Several directions for future research can be offered. Therapists’ life courses and specifically their development as professionals were previously acknowledged to be influenced by supervision processes, as well as by their experience and years in training (Messina et al., 2018). Thus, the future studies should explore the effect of the training process on therapists’ expectations and perceptions of the therapeutic process. Additional studies are needed to assess the reported associations between specific mechanisms of change and therapists’ characteristics, as well as to assess the association between therapists’ process expectations and outcomes. The findings should also be interpreted in light of the study’s limitations. As therapists in this study were psychologists who viewed themselves as either psychodynamic or integrative in orientation, the ability to generalize from the results of this study to other professionals is limited. Additional studies are needed to validate our findings with therapists from different disciplines and from other therapeutic orientations. Furthermore, as potential biases related to researcher positionality cannot be ruled out, the future studies should further validate the reported findings. Future studies should also include a larger sample so as to further confirm the validity of the findings. Finally, this study only evaluated expectations and attitudes toward mechanisms of change but did not investigate associated behaviors. Such a line of research should be performed in the future studies. Taken together, the study highlights the diverse attitudes of therapists regarding mechanisms of change in psychotherapy, as well as the correspondence between empirical research and clinical knowledge in the field of psychotherapy.

## Data Availability Statement

The datasets presented in this article are not readily available because of the need to safely ensure therapists’ confidentiality. Requests to access the datasets should be directed to DT, danatz@ariel.ac.il.

## Ethics Statement

The studies involving human participants were reviewed and approved by the institutional review board of Ariel University. The participants provided their written informed consent to participate in this study.

## Author Contributions

DT initiated and designed the study, performed the literature review, and analyzed and interpreted the data. SS collected and organized the data, and critically revised the manuscript. SA performed literature review, collected and organized the data, and critically revised the manuscript. All authors contributed to the article and approved the submitted version.

## Conflict of Interest

The authors declare that the research was conducted in the absence of any commercial or financial relationships that could be construed as a potential conflict of interest.

## Publisher’s Note

All claims expressed in this article are solely those of the authors and do not necessarily represent those of their affiliated organizations, or those of the publisher, the editors and the reviewers. Any product that may be evaluated in this article, or claim that may be made by its manufacturer, is not guaranteed or endorsed by the publisher.
