# Supplementary material for: Therapists’ Views of Mechanisms of Change in Psychotherapy: A Mixed-Method Approach
Source: Front Psychol. 2022 Apr 14;13:565800. doi: 10.3389/fpsyg.2022.565800 (PMC9050201; doi:10.3389/fpsyg.2022.565800)
Supplement: Supplementary file 1 [file Table_1.docx]

Appendix A. Full list of themes expressed by therapists, frequencies and examples

|  | Factor | Theme | N | % | Examples |
| --- | --- | --- | --- | --- | --- |
| 1 | Therapeutic bond | Therapeutic bond | 82 | 22.16% | “To evoke change there should be a strong therapeutic bond between the therapist and the patient”; “The main work is conducted within the therapeutic bond between the therapist and the patient. The ability to talk about the bond, evolve, and go through struggles within it” |
|  |  | Openness | 7 | 1.89% | “Open discussion about factors which detain change” |
|  |  | trust | 32 | 8.65% | “Sense of security and trust in the relations”; “trusting relations that enable the expression of complex contents in the room and the processing of these contents” |
|  |  | Open communication | 1 | 0.27% | “Open and honest communication”; “trust, honesty, and open communication” |
|  |  | Secure environment | 17 | 4.59% | “An open space for the patient to feel secure, hugged and accepted”; “the patient should have a secure, non-judgmental and accepting space, which enables him/her to bring him/herself and introspect” |
|  |  | Listening | 7 | 1.89% | “Genuine and deep listening of the therapist to the patient”; “Psychological listening” |
|  |  | Commitment | 1 | 0.27% | “Commitment by both patient and therapist to the therapeutic goals” |
|  |  | Devotion | 4 | 1.08% | “Willingness to devote yourself to the patient and his/her needs”; “devotion of both patient and therapist to the process” |
|  |  | Synchronization | 3 | 0.81% | “The ability of the therapist to be patient and let the patient progress at his/her own pace”; “there should be a synchrony between the patient and the therapist, so that the therapist does not try to promote premature processes, but to fit the conversation, play, or interpretation to the phase the patient is in” |
|  |  | Effort | 6 | 1.62% | “Joint effort in mutual work, needed from and in the context of distress”; “both patient and therapist should be effortful and play an active part in the therapy” |
| 2 | Therapists’ characteristics | Empathy | 21 | 5.68% | “Therapist should be empathic to the difficulties arising in the therapeutic process”; “an empathic bond should be formed to allow for expression of a broad range of emotions” |
|  |  | Curiousness | 1 | 0.27% | “Interest and true curiosity towards the patients’ distress” |
|  |  | Flexibility | 5 | 1.35% | “Flexibility which enables fitting the therapy to the patient’s needs”; “being flexible and dynamic to fit to changing needs of the patient” |
|  |  | Patience | 2 | 0.54% | “Therapist should have motivation and patience”; “the ability of the therapist to be patient” |
|  |  | Humbleness | 1 | 0.27% | “Humbleness” |
|  |  | Passion | 1 | 0.27% | “A mutual and meaningful bond that enables exploring the inner world of the patient with curiosity and passion” |
|  |  | Matureness | 1 | 0.27% | “Therapist awareness” |
| 3 | Professionalism | Professionalism | 6 | 1.62% | “Professionalism, knowledge, guidance and training of the therapist”; “broad professional knowledge”; “the therapist’s experience and the level of competence, the ability to use his/her emotions and not be scared of them” |
|  |  | Setting | 4 | 1.08% | “The guarding of the boundaries and the settings”; “the lowering of the defenses, which is enabled when there is a clear setting in the first place” |
|  |  | Knowing treatment approaches | 6 | 1.62% | “Knowledge and experience of the therapist regarding the specific problem and the appropriate treatment approaches for it”; “the use of specific therapeutic approaches” |
|  |  | Defining goals | 8 | 2.16% | “Defining the therapy as set by the goals defined by the patient and the therapist collaboratively”; “The treatment should have clear goals, which will be openly defined and discussed by both the patient and the therapist, and the treatment will be aimed towards achieving this goal” |
|  |  | Theoretical knowledge | 9 | 2.43% | “The treatment should be adapted to the patient in terms of the theoretical and practical knowledge of the therapist and the interventions used”; “theoretical knowledge about diagnoses, patient personality, treatment approaches” |
| 4 | Theory-driven  Mechanism | Desire | 1 | 0.27% | “The creation of space which enables exploration of what arises in the process of jouissance and initiating the level of desire” |
|  |  | Object relations | 1 | 0.27% | “Changes in internalized object relations” |
|  |  | Transference interpretations | 9 | 2.43% | “Appropriate management of the counter-transference”; “change in the transference and counter-transference and internalization of the therapist”; “being attentive to the transferential relations, to the enactment” |
|  |  | Mechanisms/  defenses | 7 | 1.89% | “A change can be created when the defenses are down to a point where mental movement can be produced”; “the deep understanding of patterns of defenses”; “enabling thoughts and defenses to become more flexible” |
|  |  | Experiential  processing | 8 | 2.16% | “Change cannot occur without including an experiential change, whether it is within the transferential relations in dynamic therapy or in exposures in CBT”; “the experiencing of the relations with the therapist creates a change in the personality structure of the patient”;  “corrective emotional experience” |
|  |  | Validation | 3 | 0.81% | “Validating patients’ experiences” |
|  |  | Containment | 6 | 1.62% | “A containing relationship”; “the capability of the therapist to contain the patient, in Bion’s terms, enables the patient to feel alive and with a space to introspect” |
|  |  | Self  reinforcement | 1 | 0.27% | “The reinforcement of the self” |
|  |  | Finding  meaning | 1 | 0.27% | “Finding sense and meaning in experiences in the inner world of the patient” |
|  |  | Acceptance of pain | 5 | 1.35% | “A secure encounter with mental pain”; “the ability to feel vulnerability and insecurity as part of life movement” |
|  |  | Potential space | 6 | 1.62% | “Creation and maintenance of potential space to play and think” |
|  |  | Introspection | 18 | 4.86% | “When the person gets to know the inner processes occurring within him/her and how these processes affect and shape his/her self-image, his/her thinking processes, his/her relations”; “the introspection and expansion of awareness of feelings and emotions” |
|  |  | Insight | 12 | 3.24% | “Insight towards factors which facilitate pain and create a sense of being stuck”; “having insights about the coping strategies, self-perception, and relations of the patient” |
|  |  | Emotional  experience | 5 | 1.35% | “Emotional awakening of the patient”; “exposure to emotions and the ability to correct, mourn, grow, forget and forgive” |
|  |  | Ability to play | 1 | 0.27% | “Development of spontaneity and the ability to play” |
| 5 | Clients’  characteristics | Flexibility | 2 | 0.54% | “The development of more flexible thoughts and defense mechanisms” |
|  |  | Beliefs / expectations | 5 | 1.35% | “A belief in the abilities of the treatment to form change”; “Two people who believe in the possibility of creating change” |
|  |  | Patience/sustainability | 3 | 0.81% | “Very often, just patience”; “sustainability”; “patience by both therapist and patient” |
|  |  | Curiousness | 3 | 0.81% | “Curiosity of the patient about him/herself and his/her way of being in the world”; “the appearance of curiosity in the patient towards his/her inner world” |
|  |  | Mental  resources | 1 | 0.27% | “Mental resources” |
|  |  | Matureness | 3 | 0.81% | “Level of the matureness and readiness of the patient” |
| 6 | Clients’  motivation | Motivation | 15 | 4.05% | “Motivation and willingness of the patient to meet him/herself in complex, dark and unfamiliar places”; “being ready and motivated for a change” |
|  |  | Will to change | 6 | 1.62% | “Willingness and readiness of the patient to go through a process of change”; “willingness for a change and ability to work hard in order to achieve it” |
|  |  | Readiness for change | 8 | 2.16% | “Emotional readiness of the patient”; “a combination of timing and readiness for change” |
| 7 | Cognition | Change in thought | 1 | 0.27% | “Changes in thoughts and perceptions” |
|  |  | psychoeducation | 2 | 0.54% | “Sometimes the provision of knowledge or verbally phrasing the problem” |
|  |  | Adaptive cognitions | 2 | 0.54% | “Work on adaptive cognitions”; “processes of cognitive separation processes” |
| 8 | Unable to answer /General  mechanisms | Personality  change | 1 | 0.27% | “A change in the structure of personality of the patient” |
|  |  | Understanding the client | 3 | 0.81% | “A dynamic understanding of the patient”; “the ability of the therapist to understand the origin of the patient’s distress” |
|  |  | Integrability | 2 | 0.54% | “Ability to view the situation integratively”; “the integration of contents from therapy to the patients’ everyday events” |
|  |  | Hard to answer | 4 | 1.08% | “There is no general answer to this question… I can think of so many factors and they tend to differ between therapists and between treatment approaches”; “a very difficult question” |
